# Joint Group and Multi Institutional Position Opinion: Cirrhotic Cardiomyopathy—From Fundamentals to Applied Tactics

**DOI:** 10.3390/medicina61010046

**Published:** 2024-12-31

**Authors:** Ivan Rankovic, Ivana Babic, Jelena Martinov Nestorov, Jelena Bogdanovic, Maja Stojanovic, Jovanka Trifunovic, Nikola Panic, Mihailo Bezmarevic, Jelena Jevtovic, Dusan Micic, Vladimir Dedovic, Nemanja Djuricic, Filip Pilipovic, Elena Curakova Ristovska, Tijana Glisic, Sanja Kostic, Nemanja Stojkovic, Nata Joksimovic, Mileva Bascarevic, Aleksandra Bozovic, Lewis Elvin, Ajibola Onifade, Keith Siau, Elizaveta Koriakovskaia, Vladimir Milivojevic

**Affiliations:** 1 Gastroenterology and Liver Unit, Royal Cornwall Hospitals NHS Trust, London TR1 3LJ, UKajibolaonifade453@gmail.com (A.O.); keithsiau@nhs.net (K.S.); 2 Medical School, University of Exeter, Exeter TR10 9FE, UK; 3 Clinic for Endocrinology, Diabetes and Metabolic Diseases, University Clinical Centre of Serbia, 11 000 Belgrade, Serbia; jeca.bogdanovic@yahoo.com (J.B.); natajoksimovic9@gmail.com (N.J.); alex91@hotmail.rs (A.B.); 4 Clinic for Gastroenterology and Hepatology, University Clinical Centre of Serbia, 11 000 Belgrade, Serbia; jelenamartinov@yahoo.com (J.M.N.); jelenajev89@gmail.com (J.J.); tijana.glisic78@gmail.com (T.G.); dotorevlada@gmail.com (V.M.); 5 Faculty of Medicine, University of Belgrade, 11 000 Belgrade, Serbia; dr.maja.stojanovic@gmail.com (M.S.); nikola.panicmail@gmail.com (N.P.); ducamicic@yahoo.com (D.M.);; 6 Clinic for Allergy and Immunology, University Clinical Centre of Serbia, 11 000 Belgrade, Serbia; milevabascarevic333@gmail.com; 7 Faculty of Dentistry Pancevo, University of Business Academy in Novi Sad, 21 000 Novi Sad, Serbia; drjotrifunovic@aol.com; 8 Center for Digestive Endoscopy, University Clinic “Dr Dragisa Misovic”, 11 000 Belgrade, Serbia; 9 Clinic for General Surgery, Military Medical Academy, Military Medical Academy Medical Faculty, University of Defense, 11 000 Belgrade, Serbia; bezmarevicm@gmail.com; 10 Clinic for Emergency Surgery, Emergency Centre, University Clinical Centre of Serbia, 11 000 Belgrade, Serbia; 11 Clinic for Cardiology, University Clinical Centre of Serbia, 11 000 Belgrade, Serbia; nemanjabg83@yahoo.com; 12 Institute for Orthopedic Surgery “Banjica”, 11 000 Belgrade, Serbia; filip011@gmail.com; 13 University Clinic for Gastroenterohepatology, 1000 Skopje, North Macedonia; elenacurakova@yahoo.com; 14 Clinic for Gynecology and Obstetrics, University Clinical Centre of Serbia, 11 000 Belgrade, Serbia; cara.kostic@gmail.com; 15 Department of Cardiology, University Clinic “Dr Dragisa Misovic”, 11 000 Belgrade, Serbia; neckos94@gmail.com; 16 Department of Cardiology, Moscow State University of Medicine and Dentistry, 127473 Moscow, Russia; koriakovskaia.liza@gmail.com

**Keywords:** cirrhotic cardiomyopathy, cirrhosis, pathogenesis, treatment

## Abstract

Cirrhotic cardiomyopathy (CCM) is a diagnostic entity defined as cardiac dysfunction (diastolic and/or systolic) in patients with liver cirrhosis, in the absence of overt cardiac disorder. Pathogenically, CCM stems from a combination of systemic and local hepatic factors that, through hemodynamic and neurohormonal changes, affect the balance of cardiac function and lead to its remodeling. Vascular changes in cirrhosis, mostly driven by portal hypertension, splanchnic vasodilatation, and increased cardiac output alongside maladaptively upregulated feedback systems, lead to fluid accumulation, venostasis, and cardiac dysfunction. Autocrine and endocrine proinflammatory cytokines (TNF-alpha, IL-6), as well as systemic endotoxemia stemming from impaired intestinal permeability, contribute to myocardial remodeling and fibrosis, which further compromise the contractility and relaxation of the heart. Additionally, relative adrenal insufficiency is often present in cirrhosis, further potentiating cardiac dysfunction, ultimately leading to the development of CCM. Considering its subclinical course, CCM diagnosis remains challenging. It relies mostly on stress echocardiography or advanced imaging techniques such as speckle-tracking echocardiography. Currently, there is no specific treatment for CCM, as it vastly overlaps with the treatment of heart failure. Diuretics play a central role. The role of non-selective beta-blockers in treating portal hypertension is established; however, their role in CCM remains somewhat controversial as their effect on prognosis is unclear. However, our group still advocates them as essential tools in optimizing the neurohumoral pathologic axis that perpetuates CCM. Other targeted therapies with direct anti-inflammatory and antioxidative effects still lack sufficient evidence for wide approval. This is not only a review but also a comprehensive distillation of the insights from practicing clinical hepatologists and other specialties engaged in advanced approaches to treating liver disease and its sequelae.

## 1. Introduction

Chronic liver disease (CLD) is a progressive loss of liver function as well as destruction of liver tissue, resulting in cirrhosis as its final manifestation [1]. Cardiac dysfunction is a well-known complication of cirrhosis. It includes structural and functional changes in the heart muscle, collectively referred to as cirrhotic cardiomyopathy (CCM). CCM is a significant yet often underdiagnosed complication in patients with advanced liver disease. Its epidemiology, though less studied than other cirrhotic complications, reveals a growing concern as cirrhosis-related mortality rises globally [2]. Therefore, it is imperative that we address this issue with an approach that accounts for the complex pathodynamics at play.

## 2. Definition and Epidemiology

CCM is a diagnostic entity defined as cardiac dysfunction in patients with cirrhosis in the absence of other known cardiac disorders. CCM is typified by impaired contractile responsiveness to physiological stress and altered diastolic relaxation, with electrophysiological abnormalities, in the absence of other known cardiac disorders [3,4]. Clinically, it is characterized by suboptimal ventricular response of the heart muscle to stress, which leads to the inadequate perfusion of organs. Considering its usual subclinical nature, currently there is no universally accepted and clinically implemented diagnostic criteria regarding CCM, despite modern imaging techniques [5]. Available epidemiological data is very heterogeneous: studies indicate that the prevalence of CCM in cirrhotic patients ranges from 40% to 70%, varying depending on diagnostic criteria and population studied [2]. In 2005, an initial diagnostic criteria for CCM was proposed at the World Congress of Gastroenterology following a consensus conference [6]. This has recently been superseded by the criteria proposed by the Cirrhotic Cardiomyopathy Consortium based on echocardiographic imaging parameters (Table 1) [2].

## 3. Pathogenesis

**Figure 1:**
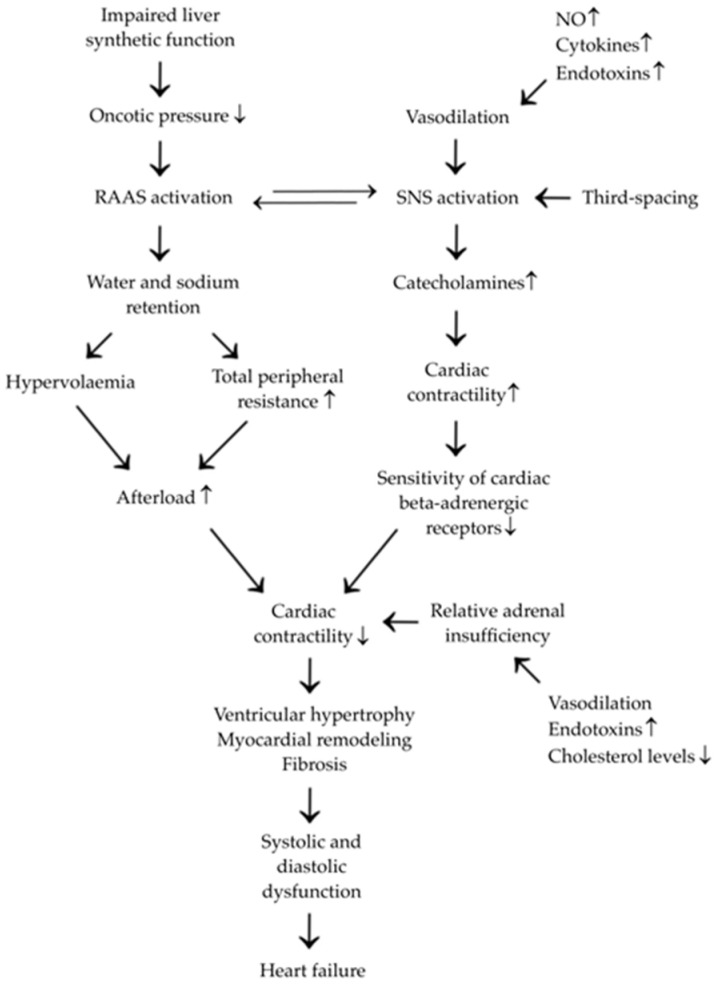
CCM pathogenesis. RAAS—renin–angiotensin–aldosterone system; SNS—sympathetic nervous system; NO—nitric oxide.

Pathogenetically, CCM appears to stem from a combination of systemic and local hepatic factors that collectively affect the balance of cardiac function and lead to its remodeling, as presented in Figure 1.

### 3.1. Hyperdynamic Changes

Cirrhosis is a condition characterized by systemic vasodilation, which is a prerequisite for the occurrence of various hemodynamic disorders in the body. Due to the hyperdynamic response of the cardiovascular system in the state of cirrhosis, mostly driven by portal hypertension, splanchnic vasodilation, and increased cardiac output, there are changes in the heart muscle function. This results in ventricular hypertrophy and eventually, systolic and diastolic dysfunction, as well as other compensatory abnormalities in the heart muscle that arise from maladaptive homeostatic mechanisms [7].

Mechanisms of cirrhosis that contribute to heart failure are based primarily upon failure of the synthetic function of the liver [8]. As fibrosis progresses, the synthetic production decreases. Pertinent to heart failure and the propagation of CCM is the drop in oncotic pressure and the intimate relationship this has with the kidneys. A drop in intravascular oncotic pressure yields intravascular volume depletion and thus activation of the renin–angiotensin–aldosterone system (RAAS) [9]. This is regulated by renal perfusion pressure, sodium levels in the ultrafiltrate of the distal convoluted tubule, intrinsic sympathetic nervous system (SNS) activity, and the negative feedback of angiotensin II (AII) on the juxtaglomerular cells [9]. In order to compensate for the decrease in vascular resistance, SNS activation occurs, i.e., heart muscle contractility increases, but water and sodium are retained through the RAAS. In cirrhosis, these feedback systems become maladaptively upregulated, culminating in increased sodium retention (causing further exacerbation of hypervolemia) and increased total peripheral resistance (further increasing afterload) [5,9]. This is especially evident during exertion and stress. Accumulation of fluid outside the intravascular and intracellular spaces (third-spacing), venostasis, and low arterial pressure upregulates the SNS primarily through augmenting the release of catecholamines. This in turn acts on cardiomyocytes and leads to a decrease in the expression of beta-adrenergic receptors, a hallmark of CCM [10]. Additionally, an increased production of nitric oxide (NO) by the failing liver, in the presence of circulating endotoxins and cytokines, and a relative state of adrenal insufficiency have further depressive effects on inotropy and chronotropy [8,10].

### 3.2. Inflammation

Cirrhosis with impaired liver function leads to the accumulation of toxins, which can have a direct effect on the cardiac muscle cells, leading to functional and structural changes [8]. Chronic low-grade inflammation is associated with compensated cirrhosis. Chronic exposure to elevated levels of circulating cytokines, such as tumor necrosis factor-alpha (TNF-α) and interleukin-6 (IL-6), plays a pivotal role in the inflammatory milieu that characterizes cirrhosis [11]. Decompensated cirrhosis further potentiates the systemic inflammation. These cytokines contribute to myocardial remodeling and fibrosis, which further compromise the contractility and relaxation of the heart due to altered cardiac metabolism as a result of impaired energy production and utilization [11]. It has been proven that myocardial fibrosis and the increase in myocardial mass that occurs during cirrhosis causes a decrease in compliance of the myocardial wall with subsequent impairment of ventricular filling [12]. Additionally, due to venous congestion, third-spacing, and intestinal dysmotility, there is an increased intestinal permeability, with bacteria and endotoxins translocating into the systemic circulation. This subsequently increases vasodilation and thus worsens the burden on the heart and the course of the disease [13]. Therefore, these patients experience the cardinal symptoms of heart failure, predominantly in the form of lethargy, dyspnea, fluid retention, and a diminished exercise tolerance. In addition, these patients also have an increased susceptibility to infections.

### 3.3. Hormonal Alterations

As a consequence of cirrhosis, various hemodynamic abnormalities develop, such as hyperdynamic vascular insufficiency, reduced peripheral vascular resistance, reduced arterial pressure, increased cardiac output, hyporesponsiveness to vasopressors, increased levels of proinflammatory cytokines, and accordingly, studies have established that endocrine insufficiency is a common cause. Adrenal insufficiency (AI) is a common comorbidity in cirrhosis [14]. It can also play a significant role in the pathogenesis and progression of CCM. Research has proven that pituitary dysfunction can be a consequence of cirrhosis [15]. More recent research has shown that liver transplantation removes these abnormalities, which supports previous studies and attests to the liver’s role in maintaining normal endocrine function. The prevalence of adrenal insufficiency in cirrhotic patients ranges from 30% to 60%, depending on the diagnostic criteria and severity of liver disease [14,15]. In this context, adrenal insufficiency is often relative, meaning that the adrenal glands fail to produce adequate levels of cortisol in response to the increased physiological demands imposed by cirrhosis [14]. This condition, known as relative adrenal insufficiency (RAI), has profound effects on cardiovascular function, including reduced myocardial contractility, hypotension, and increased susceptibility to shock.

Cortisol is the main glucocorticoid secreted by the adrenal cortex under the control of adrenocorticotropic hormone (ACTH) released from the pituitary gland. Among the factors of diurnal cortisol secretion, stress plays the greatest role [16]. During stress and serious illnesses, the activation of the hypothalamic–pituitary–adrenal axis by the action of cytokines and other factors leads to an increased secretion of corticotropin, which stimulates the production of ACTH and consequently increases the release of cortisol into the circulatory system [16]. Cortisol is a major component of cellular adaptation to stress, so an adequate level of cortisol is necessary to increase cardiac output and vascular tone and to reduce the release of proinflammatory cytokines [16].

The mechanism of adrenal insufficiency may include impaired function of total cholesterol, high-density lipoprotein cholesterol (HDL), low-density lipoprotein cholesterol (LDL), and increased levels of proinflammatory cytokines and circulating endotoxins [16]. Since the adrenal gland does not store cortisol, it must be synthesized from its precursor, cholesterol, under conditions of stress. In liver failure there is a low level of cholesterol for cortisol synthesis, thus favoring adrenal insufficiency under stress conditions [14].

Animal and human studies show that liver disease is associated with a decrease in cortisol levels and an increase in circulating glucocorticoids. In situations where the function of the pituitary gland is disturbed, and therefore the secretion of cortisol, a disturbed response of the cardiovascular system to stress results is observed [14,15]. Additionally, cortisol elimination is also impaired in cirrhosis.

## 4. Diagnosis

The clinical diagnosis of CCM remains challenging, primarily due to the lack of universally accepted diagnostic criteria and the subtle nature of its manifestations.

According to the Montreal Criteria, introduced in 2005 at the World Congress of Gastroenterology, CCM was characterized by the presence of systolic or diastolic dysfunction, along with additional indicators such as increased left ventricular mass, electrophysiological disturbances (most notably prolonged QT intervals), elevated natriuretic peptide values, and an abnormal response to stress [17].

Systolic dysfunction, in general, refers to the left ventricular relaxation impairment, described by decreased ejection fraction, usually as a result of hampered myocardial contractility [18,19]. Systolic function in patients with cirrhosis is normal or increased at rest, as a result of hyperdynamic circulation that masks the true state of the heart muscle and left ventricle. Damage to beta-adrenergic receptors and the presence of cardio-depressant substances are listed as some of the factors causing systolic dysfunction.

The characteristic of this clinical condition is impaired electrical conduction, which is reflected in electrocardiographic abnormalities, the most noticeable of which is a prolonged QT interval, which corresponds to ventricular depolarization and repolarization of the heart muscle. This finding is present in 30–50% of patients with cirrhosis, regardless of the underlying etiology and its prevalence parallels the severity of cirrhosis as assessed by the Child–Pugh scoring system [20]. Biomarkers such as B-type natriuretic peptide (BNP) and troponins have shown promise in identifying subclinical cardiac involvement; however, given their relatively low specificity due to the large number of confounding factors that can contribute to troponin or BNP elevation, their role in CCM diagnosis is still limited [21].

Considering that cardiac dysfunction may not be visible during the resting state, exercise or pharmacologic stress echocardiography is a critical tool in this regard, as it can reveal the inadequate increase in cardiac output that is characteristic of CCM [22]. Additionally, the role of advanced imaging modalities, such as cardiac magnetic resonance imaging (MRI), speckle-tracking echocardiography, and nuclear myocardial perfusion scanning, is increasingly recognized for their ability to detect early myocardial changes, including subtle fibrosis and impaired strain patterns [21].

In that sense, a revised criteria for CCM diagnosis were proposed in 2019. According to the 2019 Cirrhotic Cardiomyopathy Consortium Criteria, CCM is diagnosed when either systolic or diastolic dysfunction is detected in an echocardiography study at rest [23]. Systolic dysfunction is defined as a left ventricular ejection fraction (LVEF) < 50% or an absolute global longitudinal strain (GLS) value below 18%. The initial criteria included patients with GLS higher than 22%, but this was later withdrawn. Diastolic dysfunction criteria have been modified for patients with cirrhosis.

## 5. Treatment

**Figure 2:**
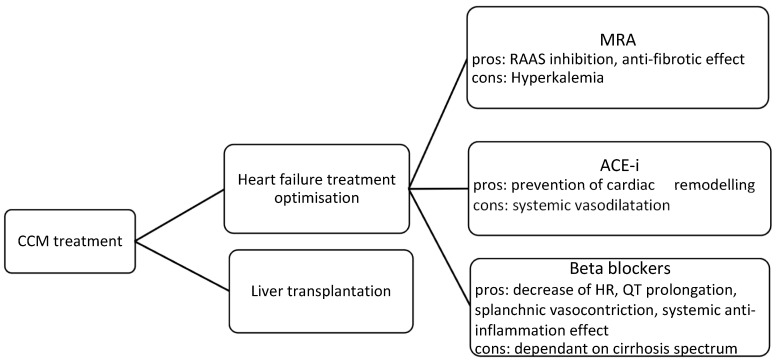
Current recommendations for CCM treatment. MRA—mineralocorticoid receptor antagonist; ACE-I—angiotensin-converting enzyme inhibitor; HR—heart rate.

When it comes to therapy, to date there is no strict treatment for CCM. When CCM progresses and produces symptoms of heart failure, patients are treated as those diagnosed with a form of heart failure (Figure 2). The heart, liver, and kidneys share an inextricable relationship with regards to the autoregulation of cardiac output. Dysfunction in any of these organs will propagate as a cycle. Considering this from the perspective of CCM, we can better understand the targeted therapies. Perturbation of the RAAS is a likely key driver in the etiology of CCM and probably underpins the morbidity and mortality benefits conferred by therapies such as angiotensin-converting enzyme inhibitors (ACE-I), angiotensin receptor blockers (ARBs), beta-blockers, and mineralocorticoid antagonists (MRAs) [24].

### 5.1. Heart Failure Treatment Optimisation

Treatment choice relies heavily on the stage of heart failure and systolic dysfunction. MRAs are indicated in patients with severe heart failure and decrease associated morbidity and mortality [25]. This is particularly pertinent considering the other advantages that these medications confer in cirrhotic patients. Hyperdynamic changes, RAAS activation, and sodium and water retention are strongly mitigated by MRAs. Additionally, it is well established that angiotensin II exerts intrahepatic vasoconstrictive and profibrotic effects; thus, the antifibrotic effect of novel MRAs can further contribute to the prevention of cirrhosis progression and decompensation [26]. Our group has found the use of eplerenone helpful due to its higher avidity with fewer adverse effects when compared to other drugs in this class.

The implementation of ACE-I remains controversial. If initiated during the early stages of CCM, they can prevent and delay cardiac remodeling. However, due to its subclinical nature, the diagnosis of CCM is usually delayed and only identified towards the latter stages. This greatly limits the usefulness of ACE-I within the physician’s armamentarium, as traditionally, ACE-Is are avoided in the decompensated cirrhotic patient due to the risk of hepatorenal syndrome and hypotension [26].

The beneficial role of beta-blockers in the treatment of CCM is multifactorial. Non-selective beta-blockers (NSBBs) reduce heart rate and cardiac output via β1-adrenergic receptor blockage, while via β2-adrenergic blockage they exert splanchnic vasoconstriction [26]. Further to this, NSBBs downregulate the RAAS by antagonizing beta-adrenoreceptors that are expressed at the juxtaglomerular apparatus. Additionally, due to the unopposed adrenergic tone, NSBBs cause a mild increase in peripheral resistance [26,27]. NSBBs have been shown to reduce the prolonged QT interval and the hyperdynamic load in patients with cirrhosis, but whether the correction of the QT interval has a positive effect on prognosis is doubtful [28]. Current guidelines indicate the use of NSBBs in primary and secondary prevention of variceal bleeding, considering their effect is predominantly exerted through the decrease in portal hypertension [27]. Our group feels that in certain settings, when QT interval responds to beta-blockade, the use of selective or semi-selective beta-blockers (e.g., carvedilol) may be of benefit. Carvedilol acts by blocking α1 adrenergic receptors and lowers intrahepatic vascular resistance. An additional beneficial effect of beta-blockers is mirrored in their ability to, via β2 receptor blockage, stimulate intestinal motility, improve gut dysbiosis, and prevent systemic bacteriemia and endotoxemia, thus ultimately exerting a systemic anti-inflammatory effect [27]. Additionally, effective beta-blockade requires appropriate dosing because of receptor upregulation and adaptation, and it is crucial to emphasize the importance of personalized dosing strategies. The “windows” of the longstanding, somewhat controversial “window theory” regarding the timing of beta-blocker implementation have now been opened to patients with compensated cirrhosis and those with small varices and uncomplicated ascites. This “expansion“ allows for the broader beta-blocker implementation in preventing and treating portal hypertension-related complications, including CCM [27].

The implementation of glucocorticoid therapy in treating CCM remains controversial since large studies evaluating its effect on CCM are lacking. A limited number of small-scale studies have mostly included patients with decompensated cirrhosis in whom CCM has not been previously diagnosed. Empirical glucocorticoid therapy in asymptomatic patients is not recommended. However, temporary glucocorticoid therapy in the state of RAI has been shown to reduce hypotension and improve vital signs, ultimately leading to a better prognosis [29]. We feel that the use of glucocorticoids is particularly helpful when liver deterioration is precipitated by infection and would suggest considering using them when heart failure in cirrhosis is coupled with sepsis.

### 5.2. Liver Transplantation

Liver transplantation remains the cornerstone treatment for cirrhotic patients. It has been shown that successful liver transplantation improves all complications and organ-related hemodynamic dysfunctions, including CCM [6]. Normalization of cardiac structure and function, as well as normalization of QT prolongation, was observed as early as one year following transplantation. However, cardiac dysfunction remains a major risk factor for perioperative management as well as post-transplantation clinical course. In that sense, preoperative clinical assessment, monitoring of the cardiovascular system during and after the operation, and proper postoperative management are mandatory in order to improve post-transplantation outcome [6].

## 6. Current Controversies and Future Perspective

Considering the multifaceted mechanisms of cardiac dysfunction in CCM and other non-cirrhotic heart disease, therapeutic possibilities are vastly limited and may not be applicable in CCM. In that sense, understanding the pathophysiological mechanism behind CCM may lead to more targeted therapies, such as antioxidants, anti-inflammatory, and anti-apoptotic agents [30,31]. We reviewed the literature for potential anti-renin therapies, specifically aliskiren, but found no positive results. Currently, research in this field is mostly limited to animal studies, rendering the therapeutic progress in this area rather slow [32,33]. The role of statins in treating CCM remains controversial, considering that, due to its subclinical nature, CCM usually becomes evident in the state of cirrhotic decompensation. However, it has been shown that atorvastatin exhibits not only lipid-lowering capabilities but also has an effect on the reduction in the inflammatory cytokines in plasma, such as TNF-α and IL-6 [34]. It also decreases the cardiac dysfunction marker N-terminal pro-brain natriuretic peptide (BNP) concentration, and it seemingly decreased cardiac chronotropic hyperresponsiveness in animal models [31,35]. Given its acceptable safety profile, it may be a good option in patients with cirrhosis except those with severely decompensated liver function. We feel statins are an attractive approach and have used them as a continued therapy for cirrhotic patients at high cardiovascular risk. Atorvastatin, being one of the most potent statins, is a primary choice for us; however, we also consider alternatives such as lovastatin, pravastatin, and rosuvastatin. In our experience, low-dose long-term statin therapy may stop the deleterious mechanisms in CCM formation. However, well-designed clinical studies are needed to fully clarify the potential benefits of the pleiotropic effects of statins.

## 7. Conclusions

In conclusion, the diagnosis of CCM, though challenging, is of paramount importance in the management of cirrhotic patients. Advances in diagnostic modalities and a deeper understanding of their clinical implications are essential steps toward improving outcomes in this population. As research progresses, CCM may transition from being a largely unrecognized complication to a central focus in the care of patients with advanced liver disease.

## Figures and Tables

**Table 1:** Direct comparison of diagnostic criteria: Montreal Consortium and Cirrhotic Cardiomyopathy Consortium.

Criteria	Montreal Consortium (2005)	Cirrhotic Cardiomyopathy Consortium (2019)
	Absence of Clinically Significant Cardiac Disease	
Systolic dysfunction	LVEF (resting) < 55%; Abnormal systolic contractile response to stress	LVEF (resting) < 50%; Absolute GLS < −18%
Diastolic dysfunction	E/A ratio < 1; TDE > 200 ms; IVRT > 80 ms	Septal E’ velocity < 7 cm/s; E/E’ ratio ≥ 15; LAVI > 34 mL/m^2^; TR velocity > 2.8 m/s
Supportive criteria	ECG: prolonged QT interval; ECHO: chronotropic incompetence to stress; electromechanical uncoupling/dyssynchrony; left atrial enlargement; left ventricular hypertrophy; Biochemical: elevated BNP, NT-proBNP, troponin	Diastolic dysfunction grading—modified ASE criteria Indeterminate grade—additional criteria: 1. IVRT 2. PV 3. Valsalva 4. atrial strain

LVEF—left ventricular ejection fraction; GLS—global longitudinal strain; E/A—peak early velocity/peak atrial velocity; TDE—tissue Doppler velocity; IVRT—isovolumetric relaxation time; LAVI—left atrial volume index; TR—tricuspid regurgitation; Septal E’—early diastolic mitral annulus velocity; E/E’—diastolic LV filling pressure; PV—pulmonary valve.
